# Experts by Experience: Qualitative Evaluation of Adolescent Participation in the Development of a Technological Intervention to Prevent Youth Suicide in Chile

**DOI:** 10.3389/fpsyt.2020.522057

**Published:** 2021-01-25

**Authors:** Sara Hamilton Schilling, Alejandra Carreño, Eric Tapia, Franco Mascayano, Romina Pitronello, Felipe Santander, María José Jorquera, María Soledad Burrone, Ruben Vladimir Alvarado

**Affiliations:** ^1^Escuela de Medicina, Facultad de Medicina, Universidad de Chile, Santiago, Chile; ^2^Programa de Estudios Sociales en Salud, Facultad de Medicina, Clínica Alemana, Universidad de Desarrollo, Santiago, Chile; ^3^Centro de Salud Pública, Facultad de Salud, Instituto de Investigación e Innovación en Salud, Universidad Central de Chile, Santiago, Chile; ^4^Escuela de Salud Pública, Facultad de Medicina, Universidad de Chile, Santiago, Chile; ^5^Division of Behavioral Health Services and Policy Research, New York State Psychiatric Institute (NYSPI), New York, NY, United States; ^6^Escuela de Psicología, Facultad de Ciencias Sociales y Comunicaciones, Universidad Santo Tomás, Santiago, Chile; ^7^Independent Researcher, Santiago, Chile; ^8^Departamento de Atención Primaria y Salud Familiar, Facultad de Medicina, Universidad de Chile, Santiago, Chile

**Keywords:** adolescence, suicide prevention, health promotion, technology, Chile

## Abstract

Adolescent suicide is a pressing problem in Chile that has not yet been sufficiently addressed, as suicide rates have stagnated in recent years. One possible explanation could be linked to the adult-centered paradigm that continues to prevail in relation to adolescent health initiatives. In light of this, programs that seek to promote youth mental health should consider incorporating adolescents in the design process using participatory methodologies, to ensure that these initiatives are well-suited for the population. In line with this recommendation, a group of seven adolescents, 13 to 20 years of age, were incorporated into a research team to actively guide the design, development, and validation of a technology-based intervention, known as Project Clan, which was piloted to reduce adolescent suicide in schools in Chile. This group was known as the “Group of Experts,” in acknowledgment of their role as experts by experience on adolescence. A qualitative case study was conducted to explore their lived experiences, through semistructured individual in-depth interviews with six members of the group. Results showed that the adolescents had a high level of interest in mental health and had experienced problems of their own or accompanied friends who were struggling, which motivated their participation in the study. They had a critical view of the previous interventions they had received through educational institutions and valued their role in the promotion of their peers' mental health through the Group of Experts. They also highlighted the importance of creating tools that complement their daily lives and provide an alternative to existing social networks, by respecting their anonymity, providing a secure place for divulgation and self-expression, and facilitating access to professional support. We conclude that programs that address issues that affect adolescents should incorporate adolescents in the decision-making and design processes to ensure the acceptability and effectivity of their interventions.

## Introduction

A central pillar of health promotion, as defined by the World Health Organization's Ottawa Charter of 1986, is the empowerment of individuals and communities to be actively involved in improving their own health, ensuring they have the power to shape their own well-being (1). This participation can take multiple forms, from helping to set priorities or planning activities to actually guiding the implementation of strategies and interventions, in collaboration with policy makers, health professionals, and other stakeholders. One noted method of community involvement is through research initiatives, and most specifically, through participatory methods, such as Participatory Action Research (PAR), which rejects the traditionally passive role of study subjects and instead views them as fellow researchers, active decision makers, and change agents (2). PAR acknowledges the value of considering individuals' lived experiences to determine how to most appropriately address issues that concern them, and in the field of health research, this approach has been used to engage vulnerable groups, such as indigenous populations or those in low-income communities, in order to conduct needs assessments, evaluate services, and establish feasible solutions to pressing problems impacting these individuals (3). Similarly, given the growing concern worldwide for youth mental health, adolescents have been enlisted in participative studies to improve the design of interventions that seek to promote their peers' well-being (4, 5).

In Chile, adolescent mental health is a major public health concern (6). According to the latest national data, nearly a fourth of Chileans between 4 and 18 years of age meet the criteria for a psychiatric disorder (7), and bullying and depressive symptoms are prevalent in Chilean schools (8). Chile is also one of the countries with the highest suicide growth rates in recent decades (9), and its adolescent population is no exception, with suicide being the second leading cause of death in this group (10). A 2010 study by Ventura-Juncá and collaborators found that more than 60% of youth have experienced suicidal ideation and that nearly 20% have already attempted suicide at some point in their lives (11). Nevertheless, fewer than half of those participants with suicidal risk were able to receive treatment from mental health professional, due to multiple access barriers. These obstacles to seeking care are not only physical—due to the lack of services or financial or time-related factors—but can also be psychological, in terms of the associated social stigma (12).

In response to this concerning epidemiological data, the Chilean Ministry of Health declared the reduction of adolescent suicide to be one of its sanitary goals for the 2011 to 2020 decade (13), and the Ministry's National Program for the Prevention of Suicide, launched in 2013, calls for the elaboration and implementation of interventions to reduce suicide risk and promote student well-being (10). Unfortunately, the real impact of these well-intentioned initiatives has been modest, and suicide rates have stabilized in recent years (9). In part, this stagnation could be due to the fact that few interventions have been implemented and evaluated in educational establishments, the frontline settings where suicide prevention programs are most needed, given the aforementioned treatment gap associated with adolescents' access to formal health services (7, 14), as well as their lack of satisfaction with the quality of these services (15). School-based interventions, meanwhile, have been shown to be appealing to students, teachers, and parents, and they significantly impact academic and psychosocial outcomes long term (16).

On the other hand, the stagnation in suicide rates could also be linked to the adult-centered paradigm that continues to prevail in relation to adolescent health initiatives (17, 18). Under this paradigm, adolescence is considered to be a preparatory period of transition before adulthood, and the specific interests and needs of this age group tend to be rendered invisible. At the same time, adolescents are apt to be defined in association with risky or disruptive behavior, such as unprotected sexual activity, drug trafficking and consumption, school desertion, and violence, and programs targeted toward this group tend to be focused solely on the early detection of risk factors and the elimination of “risky behavior” (19). While these approaches are important, they have not yet been sufficiently updated or accompanied by a more positive perspective of adolescence, which acknowledges and encourages adolescents' strengths, experiences, and knowledge, to promote their comprehensive, healthy, and safe development and overall well-being (20). The dominating adult-centric and risk-centered paradigms of adolescence are also notably at odds with the Chilean Ministry of Health's own promotion of the rights and participation of its adolescent service users, as laid out in the National Program for the Comprehensive Health of Adolescents and Youth Action Plan for 2012 to 2020 (21). Additionally, UNICEF emphasizes the urgent need to promote adolescent participation in decision-making spaces, noting the “strategic importance of this group for the development of our societies” [(22), p. 4], as adolescents are becoming mature, active citizens, who can contribute with valuable and transformative ideas. UNICEF's open call to encourage adolescent's active participation is in line with current paradigms that view adolescence as a crucial period of growth and learning (20) and with the concept of “experts by experience,” a term born out of the recovery movements led by mental health service users, which highlights the importance of valuing user' lived experiences and perspectives and allowing them to inform and guide treatment and policy decisions (23, 24). The use of “experts by experience” has also expanded beyond the field of mental health services, to encompass individuals affected by other health problems or certain social groups, such as adolescents with juvenile idiopathic arthritis or clients of long-term elder homes (25, 26).

It is thus clear that in order to best address the urgent issues of youth mental health and suicidal risk, non-suicidal self-injury (such as cutting), and suicidal behavior—as understood by the *Diagnostic and Statistical Manual of Mental Disorders, Fifth Edition* (27–29)—programs must be adapted to the adolescent population in Chile, by bringing the interventions in their regular daily settings (rather than limiting them to clinical environments) and by incorporating them as central actors in the process of designing and validating the strategies. With this context in mind, the authors applied for and were granted Chilean public research funding for a project to develop and evaluate an intervention program, using information and communications technology (ICT), to prevent adolescent suicide among students in school settings through a randomized controlled trial. At the onset of the project period, seven adolescents were recruited to join the research team to lead the development of the intervention model, as experts of the adolescent experience, in recognition of their unique understanding of their peers' interests, needs, concerns, and expectations, in relation to youth mental health, adolescent suicide, and possible interventions to address these issues. Over the course of a year and a half (March 2017 to September 2018), this group of adolescents, who were known as the “Group of Experts,” met monthly with three members of the research team (authors SS, ET, and FS) for 2- to 3-h meetings and kept in regular contact between in-person meetings via WhatsApp in order to develop the intervention model and oversee its transformation into an online web platform and mobile application, in collaboration with computer engineers and programmers, which resulted in the final version of the intervention, a virtual community known as Project Clan (“Proyecto Clan” in Spanish). The name Project Clan was chosen through brainstorming discussions with the Group of Experts, in reference to the values of peer support, inclusivity, and respect that underlie the intervention. The interventions' features range from traditional suicide prevention strategies (e.g., chat and phone line with trained mental health professionals and short articles with evidence-based information and tips), which seek to reduce the mental health access barriers, to more novel components (e.g., forums, a central “wall” to post anonymously, the creation of avatars instead of using photographs) that are focused on facilitating interactions between participants and encouraging their sense of belonging. Further details about the project and trial, which was the first of its kind in Latin America, can be found in Mascayano et al. (30).

The inclusion of the seven adolescent members of the Group of Experts in the research group added a central PAR component to the investigation, because the adolescents' ideas and proposals were the basis for the intervention model, and their active and ongoing engagement and feedback led to the creation of Project Clan. To better understand the significance and origins of the adolescents' contributions to the project, a qualitative study was conducted in parallel to the larger project and is presented in this article. Specifically, the aims of this study were to determine how the group members' past experiences related to adolescent mental health, suicide, and the use of ITCs transformed them into “experts by experience” and informed the creation of the intervention model.

## Materials and Methods

### Procedures

We used a qualitative case study design to evaluate the incorporation of adolescents in the development of Project Clan. As indicated by Stake, care studies are “the study of the particularity and complexity of a single case, coming to understand its activity within important circumstances” (31). The foremost objective of case studies is to describe and explore the characteristics of the participants and secondarily to apply this exploration to more deeply understand a specific phenomenon involving the participants (32, 33). This case study focused on the adolescents who comprised the Group of Experts during the development of the Project Clan intervention, from 2017 to 2018, to prevent adolescent suicide and promote mental health and well-being in Santiago, Chile. These cases are particularly difficult to distinguish and separate from their context, in line with Tight's description of case studies (34). Additionally, Tight adds that case studies, which are qualitative by nature, tend to rely on small samples and utilize a particular type of evidence; in this case, we use semistructured, individual in-depth interviews to study the adolescents' experiences.

The interviews were conducted by an external researcher (author A.C.), a medical anthropologist who did not participate in the development of Project Clan and who did not previously know the members of the Group of Experts. The interviews focused on three thematic areas: (a) personal experiences related to adolescent mental health (or those of their peers); (b) use and perception of online social media and social networks; and (c) experiences with and contributions to Project Clan.

The study's base assumption was that the adolescents' previous experiences with youth mental health issues and use of ICTs and social networks strengthened their contributions to the intervention model.

### Participants

The participants of this case study were the adolescents who were selected to form part of the Group of Experts for the larger suicide-prevention project. This selection process was based on two focus groups, which were held in January and March 2017, with a total of 17 participants, between 13 and 20 years of age (from eighth grade to second-year college students). Each focus group lasted ~90 min and covered topics ranging from adolescent suicide and mental health, how these issues are addressed in school settings, and the use of ICTs. On the basis of these discussions and the adolescents' gender, territorial, and socioeconomic diversity, and interest in participating in the study, a subgroup of seven adolescents—three males and four females—were selected and agreed to participate in the Group of Experts.

The specific sample for this case study was formed through a purposive non-probability method (35). On the basis of this method, the sample was limited to six of the adolescents who formed a consistent part of the Group of Experts throughout the entire process of developing the intervention model. This criterion excluded one of the female members of the group, who attended only two meetings at the beginning of the project and then had to withdraw because of academic obligations. Table 1 presents deidentified details of the six members of the Group of Experts who participated in this study. In the *Results*, the adolescents will be identified by codes, referring to their gender (e.g., [M1] is male participant number one).

**Table 1:** Group of Experts.

**Code (Gender)**	**Age (years) at time of interview**	**High school**
F1	14	Subsidized artistic school (in MR)
F2	17	Public school of academic excellence (in MR)
F3	18	Public school of academic excellence (in MR)
M1	20	Public school (in region)
M2	20	Subsidized school (in region)
M3	17	Public school (in MR)

*F, female; M, male; MR, Metropolitan Region (location of capital city of Santiago)*.

### Tools

The six members of the Group of Experts were initially informed about this study and invited to participate during their regular meetings with the research team in early 2018, and the adolescents were then contacted by author A.C. through a telephone call or via WhatsApp. In this first contact, the study and its purpose were explained in greater detail, and meeting places and times were coordinated to carry out the informed consent process and conduct the interviews. The interviews were held between April and August 2018, and interview locations were determined based on the preference of each adolescent, to optimize his/her comfort and thus facilitate data collection. Four interviews were held in the adolescents' university or school, and two were held in cafés of public libraries in Santiago.

The semistructured interviews lasted between 60 and 120 min and covered various topics in relation to the project, the conformation of the Group of Experts, the participants' personal history and association to adolescent mental health issues, and their use of ICTs. All of the questions included in the semistructured interview guide (Table 2) were previously validated by the core research team of Project Clan. Each participant was interviewed once, and the conversations were audio recorded and literally transcribed.

**Table 2:** Semi-structured interview guide.

**1. Connection with the project** • How and why were you contacted to be a part of the project? • What was your initial reaction after learning about the project? How did you feel? What did you think? • What did you know previously about topics related to youth mental health? Have you had any close experiences related to this issue? Had you talked about it before with friends or family members or in school? • What do you think about the use of technologies to promote mental health?
**2. Group** • How was the group formed? Who was a part of it, and what was your relationship like? • What problems did you talk about as a group? • What differences did you have as a group (related to socioeconomic class/school/gender/city of origin)?
**3. Adolescent mental health** • What do you think about your generation? What problems does it face? How do you view the Chilean youth of today? • How do adolescents relate with older and younger generations? (indifferences, committed, etc.) • Do you believe there is a mental health problem in Chile? Among adolescents? • What do adolescents think about health professionals? Teachers? The interventions they offer?
**4. Use of technology** • What is your relationship with technology like? What sites or applications do you use, and for how much time? In terms of your group of friends or family members around your age, what technology do they use? Which sites/apps? For how long? • What role do you think technology plays in your social and emotional life? For other adolescents?
**5. Project** • What do you think about Project Clan, the development process, and final result?

### Data Analysis

The interviews were analyzed according to the tenets of thematic analysis, a strategy that identifies thematic patterns in collected data (36, 37). Thematic analysis involves the systematization of qualitative information from a coding process that is based on previously defined categories (deductive approach), as well as categories that emerge from the collected data that are consistent with the hypotheses and thematic questions of the case study (inductive approach) (38). Thematic analysis is particularly pertinent for exploratory qualitative studies, because of its cyclical and reflective nature, which also maintains systematicity and rigor.

Authors A.C., S.S., and E.T. independently reviewed and codified the transcripts, using the program ATLAS.ti, and they met regularly to compare and discuss their analyses and emerging categories and review any discrepancies. Through this iterative process, they arrived to a consensus about the final categories and the principal findings.

### Ethical Considerations

Prior to beginning each interview, the participants—and in the case of minors, their parents or guardians—went through an informed consent process, in the context of the larger project, which was approved by the Ethical Committee of Human Subjects Research of the Faculty of Medicine, Universidad de Chile. The adolescents did not receive any sort of payment or reward for participating in the study, and any incurred costs (transportation, beverage) were covered by the project or reimbursed, to ensure that their participation did not represent a financial burden.

## Results

The categories used to analyze the data coincided with the three initial thematic areas of the case study. In addition, a fourth emergent category was added, dedicated to the analysis of other mental health interventions that the adolescents had been exposed to previously. These different interventions assumed a central importance in the participants' explanations of their past experiences, as they noted that these interventions motivated their participation in the Group of Experts and informed their vision and principal recommendations for Project Clan.

These four main categories are as follows:

- Relation to and experience with mental health
- Prior interventions
- Relationship to social media and social networks
- Reaction to and experience with Project Clan

Main findings of each of these categories are detailed below and also summarized in Table 3.

**Table 3:** Main findings of the study by category.

**I. Relation to and experience with mental health**	**II. Prior interventions**	**III. Relationship to social media and social networks**	**IV. Reaction to and experience with Project Clan**
1. Based on their experiences with their peers, the adolescents wanted tools to prevent and help their friends and classmates with mental health problems.	1. The mental health interventions that have been offered to the participants in school settings were considered to be “adult-centric” and incongruent with their social and economic realities and family contexts.	1. While the participants constantly used social media, they were also aware of the associated risks, in terms of privacy invasion and the addictive nature of the platforms.	1. The adolescents thought it was necessary to create a platform and interventions that were different from the social media platforms and networks that already existed, which motivated their participation in the project.
2. Based on their own experiences, the participants expressed wanting to more deeply understand their own changing feelings and emotions related to adolescence.	2. The participants recognized an access gap between adolescents and mental health services, due in part to persisting stigma.	2. The adolescents said they had utilized strategies to protect themselves from these risks, by regulating their use and reporting inappropriate behavior.	2. The adolescents recommended the construction of a personalized, visually attractive platform that protects their anonymity, through the use of avatars, for example. They also advocated for the incorporation of mental health professionals (“Clan Councilmembers”) to reduce barriers to access mental health services.

### Relation to and Experience With Mental Health

This thematic category focused on how the participants learned about adolescent mental health issues, through their own experiences and those of their peers. These experiences pushed them to want to acquire tools and competencies to effectively help others in times of need.

#### Peers' Experiences Driving Participants' Desire to Help

The adolescents involved in Project Clan had a high level of interest in topics related to mental health and had personal experiences to share pertaining to their own mental health struggles, their friends', and/or those of another close contact. Each participant, without exception, gave detailed accounts about the various ways in which they have seen their school-aged years impacted by mental health issues, from bullying in classrooms or on social media, the problematic use of drugs and alcohol, to the presence of depressive and anxiety symptoms linked to family problems and the acceptance of sexual identity, as well as cases of self-cutting and suicide among their classmates. In the face of these experiences, most of the participants positioned themselves as peers: “When you know about these things, that a friend, a classmate is suffering, you want to help, to say something that could help him or her feel better” [M1].

Nevertheless, the interviewed adolescents admitted that, although they wanted to support their peers, they did not have adequate skills, tools, or training to meaningfully offer any sort of assistance:

[I had a friend that cut himself, on his arms, and] I always told him that he didn't have to do that and that he had to realize that he had friends who were there and cared for him, but more than that, I didn't know what else to do… the problem is that I only knew about what was happening because my friend trusted me, and I wasn't about to go to telling everyone, so I just tried to help him however I could, but for me, it wasn't justified to go tell a teacher, because then I would be sharing things he didn't want others to know. [F1]

The adolescents shared that, although they were frequently witness to their friends' mental health problems, they did not know how to adequately address these issues, apart from accompanying their friends and giving advice to the best of their ability. They also expressed feeling impotent, as they were unable to resolve the situation:

You knew about bullying, that people bugged your friends, but you didn't really know what to do. Everyone wants to fit in, so you try not to worry too much about it, and that's it. I hated seeing this, and at least I didn't react this way, but it bugged me… But, actually, the truth is, I didn't do much. I didn't know what to do. [F2]

The participants' desire to have been more prepared to help, while still respecting their friends' request for secrecy, was a significant part of their motivation for participating in the study and forming part of the Group of Experts to create Project Clan. As one participant explained:

Suicides were common at my school, and I had some friends that also seemed to be heading in that direction. [With Project Clan,] I really liked that I could somehow help them, to get them to trust me and share their problems, so that I could help solve them or at least help my friends keep going and see that everything can get better. [M2]

#### Personal Experiences and Desire to Understand Their Own Growth Process

Likewise, the participants' own experiences, having overcome difficult moments related to depression, cutting, anxiety, and bullying, also motivated them to help their peers.

When they told me about the project, I was interested because I know about mental health. I've been going to see psychologists, psychiatrists, everything, since I was about 10. They had me read things, to learn about it, and everything was lame and boring. They had me answer surveys, tests, and I always wondered what it was good for. “I'm still sad, this isn't working,” I thought. So, when they told me about a platform where I could play, keep track of my mood, and talk with other people, I thought that it could be exciting, even for someone who doesn't have problems. It's not a typical site run by psychologists. [F3].

In this same vein, another participant who went through a similar process during his adolescence shared that he wished that he would have had access to something like the project's platform to support him when he when he felt alone and helpless.

I was bullied when I was younger, and that experience affected my mental health during high school. That is what got me to contribute to the project and help… Because during that period, it was like… mistake after mistake. I told the school administration what happened and nothing. In fact, they told me “hit them back.” In the end, I was alone… But that could have been completely avoided. Over time I've come to understand that people have choices, and if for example, a kid bullies others, there's some reason behind it. Maybe his parents abuse him, maybe his parents are separated and fight every day, but I only came to understand that when I grew up and matured. It could have been avoided. [M1]

The participants' testimonies reveal their familiarity with a range of issues that are central to adolescent mental health. Moreover, their discourse displays how these topics have become an integral part of their daily lives and interactions. As they constructed their identities, the participants' views on mental health evolved and matured, and they gained new tools to help them overcome obstacles. With Project Clan, they were motivated to share their collective and personal experiences and knowledge to assist their peers.

### Prior Interventions

This second category, which emerged from the first thematic area, is related to the adolescents' experiences with prior intervention programs and activities they had seen or participated in through their school or other settings, as well as their reflections about the traditional approaches to youth mental health promotion in Chile. Their opinions of these interventions influenced their proposals for the platform, which sought to remedy some aspects to the approaches they considered to be most critical and at fault, such as an adult-centric and risk-centered perspective of adolescence, their interests, and issues; and indifference about the sociofamiliar contexts of students. Likewise, these reflections reflect that the adolescents felt removed and distanced from traditional mental health services, in part due to stigma about the use of clinical services.

#### Drawbacks of Adults-Centric and Risk-Centered Interventions

The participants had a deeply critical view of the few, if any, mental health interventions they received during middle and high school, from teachers, school counselors, or administrators. Despite their peers'—and their own—psychological struggles, the adolescents reported that some of their schools showed almost complete indifference to their difficulties, as did most of the adults in their lives. The Chilean education system is heterogeneous, and the quality of education received depends largely on socioeconomic level, with some schools strongly shaped by market interests, rather than the educational needs of the population (29). In this context, the participants' schools had varied responses to the students' mental health issues. While some students participated in workshops about bullying and drug use, others shared that they did not receive any mental health orientation, be it during classes or through extracurricular activities. Despite these differences, the participants said that all of the interventions were offered through an adult-centric lens, with professors simply providing them with information to correct the students' supposed ignorance about substance abuse and other “risky behaviors.”

In my school, they occasionally tried to do things like, I don't know, an antidrug program, because you could often see people using or selling drugs inside the school, so they organized these super inadequate programs, led by someone who had never used drugs in his life. I think that someone who has never used drugs isn't going to be able to help someone who uses, because he's never been there. What's more, they brought in a sociologist to talk to us, who was clearly very upper class. It was like he came from this completely different, comfortable reality, and comes down to study us poor students. Like, “let's find them a solution.” But it was from a position of absolutely zero experience, and so obviously just listening to him made you fall asleep (…). So the programs the schools have are so deficient that it's funny but also sad. [M3]

The participants also explained that, because of their schools' deficient strategies for addressing these important topics, they did not feel comfortable reaching out to adults when there was a problem. They stated that they believed that their teachers do not have the tools or time to adequately assist them. One participant explained that his teachers' failure to respond to bullied classmates made the classmates downplay what they were going through and even believe that the bullying was not an issue.

The teachers knew [about the bullying] but did nothing. A few times I tried to do something, but then I realized that even the bullied kids, when you talked to them to try to ask how they were or tell them to do something, even they would tell us, “nah, I don't care,” like playing dumb. “We were just playing. It doesn't matter.” Things like that. So, it just went on and no one intervened. [M2]

At the same time, they feared that if they opened up to their parents about their problems and crises, they would be viewed as problematic or “crazy” and be sent to a clinical psychologist, which they viewed as another obligation that would only serve to “increase the pressure [they] are under” [F2].

#### The Gap Between Adolescents and Traditional Mental Health Services

The treatment and support access barriers facing adolescents, marked by their mistrust and rejection of the schools' insufficient efforts to address important issues, are further increased by the stigmatization of mental health services. The participants expressed skepticism about how health professionals and other adults could help them, and many of them shared that while in school, they avoid talking with these individuals about their or their friends' issues, even when faced with serious problems.

There are people that don't show how they feel. They seem happy, but inside they are not okay. But these people, these students, are afraid of showing how they really feel, because when they tell you “go talk to the psychologist,” it's scary, because you think “What are they going to tell me? What are they going to ask?” and so people don't go, even if they feel really bad. There's the option, but they are not going to take it, because they feel like it's going to be weird, and make them feel worse. [F1]

This same participant also expressed serious doubts about the effectiveness of traditional mental health services:

I had a friend who was cutting himself, and I never told his mom because I thought his mom was going to hospitalize him (…). He always told me that it was a good idea not to tell his mom, because she would have done tons of things that wouldn't have helped him… It wouldn't have been worth it to spend money on a psychologist. [F1]

### Relationship to Social Media and Social Networks

The participants' use, perception and relationship with ITCs, and specifically online social networks, revealed the adolescents' awareness of the benefits of these networks but also the risks inherent in the use of these platforms. They also valued the importance of anonymity in seeking support on mental health issues.

#### Benefits and Risks Related to Social Networks

The adolescents indicated that mental health promotion should be undertaken through an intersectoral collaborative approach, while avoiding vertical, risk-centered interventions that do not address adolescents' interests and realities and that underestimate their experiences. Additionally, they agreed that digital resources, in particular social networks, could be a strategic tool on which to base interventions targeted toward their peers, given the prevalence and familiarity of their use.

In this respect, during the interviews, the participants stated that technology was a fundamental part of their social life, and they expressed frequently using technology to form and sustain relationships. All of them had accounts in the social network applications most commonly used in Chile, such as Facebook, WhatsApp, Instagram, and SnapChat, and reported using these applications for multiple hours each day. In fact, WhatsApp was a means of communication used to contact the adolescents to coordinate these interviews. It must be noted that the adolescents recognized the risks involved in the extensive and unregulated use of technology, its centrality notwithstanding, in terms of its prejudicial effects on both physical and mental well-being. With respect to the latter, the participants acknowledged that the excessive use of social networks can damage self-esteem and their ability to form long-lasting bonds. For example:

The thing is, nowadays, people do things that aren't necessary, that don't make any sense. Like, now, for example, if I take a picture of us drinking coffee in the interview, I feel like that's unnecessary. Why does everyone else have to know what I'm doing? And I think that this sort of attitude just makes us worried about other people think about us, and also, we tend to worry a lot about the lives of everyone else. [M3]

The probability that social networks have a negative impact on adolescents seems to depend on how much weight and importance teenagers give them, while forming their identities and worldviews.

I think that if we did a survey, we'd find that everyone is concerned about what other people think of them, and this can really impact your life, because sometimes people take it poorly, and begin to feel even worse and harm themselves, since they only believe and care about what other people think, and they even forget their own view of themselves. So, a lot of people get messed up because of this, because of other people's comments in social media. [F1]

#### Protection Strategies Related to Use of Social Networks

In response to this risk, a number of participants, especially the females, discussed ways in which they have protected themselves and their classmates against the negative effects of social media:

In my school, there was a problem with bullying associated with a fake page that some people made, which said horrible things about everyone, and so we went to talk to the head teacher, who asked us what we were going to do to fix this. So, we repeatedly reported the page until it was shut down for offensive content. We organized together, and it worked. It shows that when something or someone gets out of line, we can do something about it. [F1]

This type of bullying, or similar risks, such as the possibility of being tricked or contacted for inappropriate reasons, are directly related to the use of social media, which is why the participants believe that the use of social networks should be regulated especially during early adolescence (10 to 13 years), the period in which they admitted to first using these applications. As one female participant explained:

I was only allowed to begin using social networks when I was 12, and I was always supervised. (…). My mom controlled my phone, and I'd obviously say “Oh Mom, don't mess around in my life,” but now in hindsight I feel that it was the right thing, because even now sometimes people I don't know talk to me, or use my photos to create a fake profile, or I people sent me semipornographic photos. [F2]

The participants' awareness of the benefits and potential of digital resources and social networks, along with their associated risks, put them in a unique position to contribute to the creation of the technology-based intervention model to promote adolescent mental health and prevent suicidal behavior.

### Reaction to and Experience With Project Clan

The final category focused on the adolescents' initial impressions about the project proposal, the formation of the Group of Experts, and the contributions they made to the design of the intervention.

#### An Online Platform to Promote Mental Health: A Different Approach

With regard to this last category, the proposal of an intervention based on an online platform solicited diverse initial reactions. On the one hand, some of participants reported thinking that it could be “fun” and “different” and that it could be useful and interesting for their peers. These appreciations were based on the participants' impression that currently existing online material about mental health is purely informational and not helpful: “They tell you not to kill yourself, but that doesn't really mean anything for you” [F3].

A number of other participants, however, were at first doubtful that the new platform could offer something not already available through preexisting and popular social networks, in terms of forming connections and interacting with others.

At first, I thought that there are thousands of social networks. It has to have something that really attracts people's attention, or else it isn't going to work… plus, it's about a topic that people don't normally talk about openly, and presenting that information, even if it is done in a nice way doesn't automatically mean that people will use it. [F2]

The adolescents' awareness of the challenges involved in creating the online intervention model served as a starting-off point from which the group addressed issues and made decisions related to the platforms' proposed components and strategies. Moreover, the participants viewed an opportunity to develop a space that would promote, rather than weaken, their peers' mental health. They felt that current social networks facilitate unhealthy comparisons and often lead low self-esteem and loss of self-worth:

I see it a lot among girls that they compare their photo to someone else's, think about if they have a good body. If they go to the gym, they take a picture, and then someone else compares herself and feels bad. It's all really false, and we have become so dependent on that. [M3]

#### Recommendations: The Importance of Design, Anonymity, Peer Support Options, and Readily Accessible Mental Health Counseling

Numerous challenges emerged during the creation of the intervention model. The participants believed that the imagery and styling of the site would be crucial to appeal to their peers and discussed how to balance designing a visually attractive and dynamic platform that would be able to maintain the interest of adolescents, while also promoting their mental health, by strengthening protective factors and identifying risk factors.

When we were planning it, I said that it's necessary to give the users the power to change the format of the platform. It gives a greater sense of ownership… of “Oh, I'll change the color” …it makes it more personal for me, because depression is already a super personal thing… having something that is also nice to look out, without someone many weird words and messages like “you must do this” and also with colors that are more calming than red, blue, and yellow. [F2]

To astutely address these issues, the Group of Experts also decided that anonymity of the participating adolescents should be a central characteristic of the site.

I think it's great [the anonymity], because then you remove the prejudices involved in looking at a photo, when you say, “Oh no he's fat, he's ugly, or he's trashy, so I won't talk to him.” So, with anonymity, you take away that prejudice due to physical appearance, which is the first judgment one makes. [F2]

The participants' awareness of the importance of anonymity is due precisely to their interactions with one another in the Group of Experts. As that group is heterogeneous, they began to ask themselves about the opportunities that Chilean adolescents in this day and age actually have to get to know different realities and, at the same time, talk about their common interests and experiences they share as teenagers.

We got the idea of using anonymity because we looked at each other and asked ourselves, “What would you think if I someone like me talked to you, for example?” And the other person would say, “It'd be really weird, because it's not like we have a similar style,” and then I had to admit that I'd probably act the same way, too. “I'd also think it was strange if someone like you, with your physical appearance, talked to me.” So, it was like, and if we make the platform anonymous? And then we decided to do it. [F2]

However, even though anonymity has its benefits—permitting free expression and facilitating new connections by avoiding the stereotypes imposed by photographs and physical appearance on social media—the participants also realized that it was essential that images be somehow incorporated into each person's profile, as self-representation is so important to adolescents, especially in digital platforms: “It was hard to imagine a platform without images. Today, everything revolves around images, publicity, television, series, Instagram. We are also saturated with images. How are we going to get people to use the site if there aren't images?” [M2]. To respond to this need, the participants proposed using avatars or virtual characters that each person could create based on his/her interests:

We created elements for the avatars that couldn't be distinguished based on gender or age, so that everyone could identify with the character. The idea was to be as inclusive as possible and that people could choose characteristics according to how they felt or wanted to be represented. [F2]

Through the creation of avatars, the Group of Experts sought to reconcile the importance of anonymity with adolescents' need for personalization. It was also decided that the possibility of writing completely anonymous messages on a central public wall—without even the avatars linked to one's comments—would be attractive to users who want to express themselves without fear of judgment.

Similarly, the participants recommended including a gamification aspect in the platform by awarding active participation and progress in the intervention's activities with points, as a way to encourage sustained use of the platform. This idea was proposed by members of the Group of Experts who had previous experience with video games, in which players gain points as their advance and compete against each other Although the promotion of competition between the platform users was initially debatable, all of the participants agreed that the point system would stimulate use, adherence to, and interest in the platform, through a compensation mechanism, in which increased participation would unblock elements for the avatars.

Yes, I think that [the use of earning points for participating] can work, because these points could generate and incentivize a sort of competition, which is linked to progress. You could have conversations with others to ask them like, “And you, what did you do? How many points do you have?” And they listen, and in the end, there is this shared understanding, and everyone wants to advance to the next level. [M1]

They voiced that they appreciated that that their level of success and progress in the platform depended on them and their degree of engagement, and not on the effect their actions had on others, in direct contrast to the basis of their interactions in other sites, in which they write a tweet or upload a photo to Instagram or Facebook, and then view their success based on how many “retweets” or likes they receive.

Additionally, the participants' past experience viewing traditional clinical services as sterile, stigmatizing, and removed from their reality informed their recommendations that the adolescent users should be able to have rapid, easy access to trained psychologists—known as the “Clan Councilmembers”—who they could engage with through a private chat system or more publicly in different public forums with multiple participants. They believed that facilitating the online connection would make their peers more apt and open to seek out help, and that the online presence would serve to create a more horizontal therapeutic relationship. As expressed by M1: “we had an idea that a psychologist should be available 24/7, to pay attention to these problems, who you could always talk to, without being ashamed.”

Finally, during the course of developing the Project Clan model, in line with their expressed desires to help their peers (see Section 3.1.1), the participants advocated for the inclusions of functions that would facilitate communication and peer support between adolescents, such as forums, as well as informative sections about how adolescents can help their friends, classmates, and other acquaintances with mental health issues or other problems.

Thus, based on the Group of Expert's recommendations, the Project Clan platform offered an attractive, secure, and completely anonymous space for free expression, while also promoting the creation of strong social and therapeutic relationships.

## Discussion

The objective of this case study was to determine how the adolescent members of the Group of Experts' past experiences related to mental health, suicide, and the use of ITCs transformed them into “experts by experience” and informed the creation of the Project Clan intervention model to promote their peers' mental health and well-being and reduce suicide risk. The results of the four identified categories—the participants' past experiences with mental health, their experience with prior mental health interventions, their relationship to social media and social networks, and experience with Project Clan– are discussed at continuation.

Firstly, in their testimonies, the adolescent participants expressed great interest in mental health and their concern for the well-being of their peers. They shared that during high school, they were often confronted with difficult situations affecting their mental health or their friends'. However, despite wanting to find solutions to these problems, they felt unequipped to adequately respond to these important issues, due to their lack of knowledge and resources, and their being naturally assigned a passive, secondary role in the management of their own well-being. As a result of this, they felt impotent in the face of what they viewed to be a major concern for their generation. In contrast to this frustrating situation, their participation in the “Group of Experts” gave them a platform from which they could finally channel their desire to effectively help their classmates (17). Their prior experiences of feeling helpless and ignorant informed the Project Clan model, with its focus not only on one's own struggles and growth process, but also on gaining the necessary tools and information to assist their peers, by knowing how best to identify and help friends in need. These findings are in line with the central role that peer groups play in adolescent psychosocial development (39); during adolescence, one's peers greatly influence the crystallization of identify and emotional regulation, and there is an important association between social support and subjective well-being among adolescents (40). This is also highlighted in their recommendation of including forums and a messaging system in the platform, to offer a space for participants to support one another, in a safe community environment.

These components of the platform, and the formation of the Group of Experts itself, is related to the ideals of peer support, which is “based on the belief that people who have faced, endured, and overcome adversity can offer useful support, encouragement, hope, and perhaps mentorship to others facing similar situations” [(41), p. 443]. Peers are defined as individuals who have shared difficult, similar experiences, which could vary from cancer survival, to the unexpected death of a parent, to mental illness (42). In the context of Project Clan, with its focus on suicide prevention and mental health promotion, the Group of Experts shared the experience of going through middle- and high-school, facing the related stressors and pressures, and having similar goals and interests as the study's participants; on the basis of this experience, they offered mutual support and orientation. Thus, Project Clan is constituted as a peer support model. Several reviews have noted that peer-support services are of use to connect individuals with mental health issues with others, so that they can share experiences and provide emotional and social support (43–45). Furthermore, being involved in such services is often related to improved empowerment and increased hope in the future among peer workers (44). We must note, however, that the evidence of the effects of these services is still inconsistent across studies (46) and future research needs to examine the mechanisms through which peer-based interventions work.

Secondly, with respect to past mental health interventions the participants experienced, they shared that the few programs they had been offered tended to be vertical and adult-centric, thereby confirming the findings of the reviewed literature, which discussed the dominant prevalence of the adult- and risk-centered paradigms of adolescence (17–19, 47). The participants stated that the interventions they had previously seen did not comply with the criteria they considered essential for the acceptability and relevance of an intervention model (48, 49), and they also viewed traditional mental health services as distant, stigmatizing, and ineffective, reflective of the documented treatment gap between adolescents and clinical services (7, 14). Our results show that the adolescents wanted to ensure the creation of an intervention which respected and understood their point of view, which could be seamlessly inserted into their daily lives and which nurtured the establishment of horizontal, rather than vertical, caring relationships, with peers and professionals, thus reducing access barriers.

Thirdly, in terms of their relationship with technology, the adolescents acknowledged the central role that online social networks play in their daily lives, as evidenced in their frequent use of these platforms, which they use to establish and maintain their interpersonal relationships. This is reflective of the Chilean context, where over 90% of youth connect to the internet at least once a day, especially to social networks (50), and where adolescents view online resources as an important facilitator for numerous aspects of their lives (51). The participants envisioned Project Clan as a platform that had the potential to span and connect their cyber relationships with their offline, in-person activities, more so than the social media networks they currently use. At the same time, during the interviews, the participants were able to identify risks associated with the use of ITCs –from privacy concerns to mental health problems due to online bullying, abuse, oversharing, and constantly comparing oneself to others– and they shared strategies they have used to reduce these risks, which they also translated into recommendations for the intervention.

The adolescents' reflections are also supported by the existing literature. On the one hand, extensive use of social networks can lead to dependence and distress associated with anxiety and loneliness among adolescents, and there are legal and privacy concerns associated with uncontrolled use of these platforms; on the other, ITCs offer enormous potentialities for engaging adolescents and reducing access barriers to mental health services, by removing the physical distance to access quality information and orientation from mental health professionals, and by providing this support at little or no cost, all from the privacy of one's phone or computer, without the associated social taboos (52–55). The diverse and varied impact of ITC use, over the short- and long-term, needs to be intensively and continuously investigated, and to this end, there is an important, growing field of qualitative research which explores, for instance, how the use of ITCs, such as online blogs, can pose both risk and protective factors for suicidal and self-injurious practices among adolescents (56, 57).

Finally, on the basis of the above, the participants were able to capitalize on and relay these personal experiences and reflections to their role as “Experts by Experience” to create the Project Clan intervention model. They viewed Project Clan as an opportunity to contribute to a new type of online platform, which would provide a space for adolescents to find reliable information and practical advice related to mental health, share experiences, find peer and professionals, and strengthen their mental health, in contrast to existing sites they cited as harmful. The adolescents made a series of recommendation to ensure the platform's aesthetic, components, and functionality were attractive and useful for their peers, and one of the most crucial aspects for them was protecting the future users' anonymity, which would allow users to interact with others, explore new interests, and express themselves, without the fear of judgment or prejudice. Another key element is related to the centrality of adolescent self-representation and their need to create a social media identity, just as they are in the process of forming their own identity as maturing individuals in society (20, 55). The participants were able to satisfy both these needs—self-representation and anonymity—through their idea of creating avatars. Moreover, based on their desire to be able to help their peers, they incorporated spaces, such as forums, in the platform for the adolescent users to offer one another advice, in addition to the information targeted at giving tips to classmates and friends of those in needs. These strategies are in line with the literature that demonstrates the importance of peer groups and social support for adolescent well-being, which is a protective factor for mental health problems and to prevent bullying, for instance (40, 58, 59). Lastly, they viewed the psychologists—the so called “Clan Councilmembers”-–who would be available to give users individual and group orientation and support through the platform, as a positive contribution, which would serve to reduce treatment access barriers in Chile (7, 14).

### Limitations and Future Studies

The establishment of the Group of Experts to create the Project Clan model is a first approach to putting into action the Chilean Ministry of Health and UNICEF's calls for increased adolescent participation in decision making spaces (21, 22). Unfortunately, the project, and thus the Group of Experts, was only temporary, and this study also has some associated limitations. For one, given that the adolescent participants were recruited on the basis of their involvement in a specific and time-limited initiative, the study is not entirely replicable, and the results of our case study should be interpreted as a documentation of the members of the Group of Expert's unique lived experiences and their relationship and application to Project Clan, which sought to promote adolescent mental health in Chile. Second, while we hope that the findings could be applied to promote the benefits of adolescent participation through similar interventions in the future, the generalizability of these findings and the experts' experiences to the general adolescent population is not clear; the study sample was formed based on a purposive non-probability method (35), recruiting adolescents who participated in the Group of Experts and were chosen for that group because of their particular motivation and interest to participate in the research project; their views do not necessarily represent those of their entire peer group. Finally, because the Group of Experts disbanded once the intervention was launched in schools, the long-term impact of the adolescents' experiences participating in the development of the Project Clan intervention model on their own well-being and future conduct and undertakings is unknown. Future research to evaluate adolescent participation in projects and programs should have a longer follow-up period, to study possible long-lasting effects, both on the adolescents and on the eventual recipients of the programs.

Furthermore, measures should be taken to ensure the permanent incorporation of adolescents in established organizations dedicated to addressing issues that concern this age group (from health or education to recreational interests) or that will impact them as they age (e.g., climate change, labor and retirement policies, among others). Current legislation in Chile does not recognize this progressive autonomy of adolescents, to allow them to organize before they legally come of age, and there are few institutional mechanisms in place to monitor adolescent participation in the construction of public policies and the protection of their rights, in accordance with UNICEF's recommendations (22). Mere consultancy roles are not representation, and involvement in time-limited, specific one-time initiatives, such as with our project, is not sufficient. Future initiatives—from research studies to committees and executive boards—should actively promote real and effective adolescent participation (22, 60, 61).

Finally, although this is not a clinical study, there are also possible clinical applications of the Project Clan initiative. The “Experts by Experience” approach could be applied through the incorporation of peer workers in mental health services. In the words of psychiatrist and peer support advocate Peter Stastny, mental health peer support is based on the idea that individuals who have “experienced and overcome a particular type of adversity can be useful sources of support, encouragement, and help to others experiencing similar situations” (62). Peer workers have been applied in a variety of clinical interventions, for an array of psychiatric conditions, from substance dependence to depression to psychosis (63). Peer support services are widely spread and accepted in high-income countries (64), although, unfortunately, peer workers are rarely involved in planning or providing mental health care in lower-income settings, such as Latin America.

One exception is a study we conducted in Santiago, Chile, which incorporated peer workers into a community mental health intervention, called Critical Time Intervention—Task Shifting (CTI-TS), to support users with schizophrenia (65). A qualitative study that evaluated the users' experience with CTI-TS found that the users felt a special connection and reciprocity with the peer workers, who had also experienced psychosis (66). One user commented that being with the peer worker was like “talking with someone who get and who gets you, with needing to talk or explain much,” whereas another said, “I felt reflect in the peer and tried to be like her, because she went through a situation like mine; she was always smiling, and didn't seem to be someone with problems, and so seeing her doing so well gave me strength.” We believe that adolescents who have had mental health problems, including suicidal conduct, and recovered can be a powerful source of support and orientation for their peers who are struggling with similar issues, and these connections can extend from innovative public health programs such as Project Clan to outpatient mental health centers, through the establishment of support groups, mentoring, and peer support services.

## Conclusions

Project Clan is one of the first initiatives in Chile to actively include adolescents in the central decision-making processes and creation of a program that aims to improve the mental health and well-being of their peers. The adolescents involved in this project were recruited as “experts by experience” of their peers and treated as equal collaborators alongside investigators, in the entire process of creating and designing the intervention model. This is in contrast to their usual consultancy role in other programs, which tend to invite adolescents to participate in focus groups to reflect and comment on already prepared products, rather than have them brainstorm and design the tools themselves (4). The integration of adolescents in the early stages of the study permitted the transformation and translation of their experiences into an intervention model that responds to their needs, visions, and expectations for suicide prevention and mental health promotion (3).

Project Clan offers an intervention model that breaks with the traditional paradigm of mental health services, which tend to be vertical, face-to-face, solitary, and removed from daily life and in which adolescents have passive roles, as patients in a clinic or the audience of an adult-centric or risk-centered presentation or workshop. The Group of Experts channeled the adolescents' lived experiences to create a model that brings effective tools and support into the ICTs that accompany them in their daily lives, while also promoting the creation of a safe and inclusive community. The incorporation of the adolescents as a PAR component in the development of Project Clans offered the target population the agency to actively impact and improve their own well-being and that of their peers by sharing their expertise. The experience indicates that by opening up true decision-making spaces to adolescents, stakeholders can help ensure the appropriateness of their efforts for this important and increasingly empowered sector of the population.

## Data Availability Statement

Transcripts (in Spanish) of the interviews conducted for this study are available upon reasonable request to the corresponding author.

## Ethics Statement

The studies involving human participants were reviewed and approved by Ethical Committee of Human Subjects Research of the Faculty of Medicine, Universidad de Chile. Written informed consent to participate in this study was provided by the participants' legal guardians.

## Author Contributions

SS, AC, ET, FM, MB, and RA contributed to conception and design of the study. SS, ET, FS, RP, and MJ worked closely with the Group of Experts. AC conducted the interviews and carried out the initial analysis. AC and SS wrote the first draft of the manuscript. ET, FM, and RP contributed to sections of the manuscript. All authors contributed to manuscript revision and read and approved the submitted version.

## Conflict of Interest

The authors declare that the research was conducted in the absence of any commercial or financial relationships that could be construed as a potential conflict of interest.
